# COVID-19: interpreting scientific evidence – uncertainty, confusion and delays

**DOI:** 10.1186/s12879-020-05387-8

**Published:** 2020-09-08

**Authors:** Julian W Tang

**Affiliations:** Department of Respiratory Sciences, University of Leicester, Leicester, LE1 7RH UK

One of the emerging aspects of the current severe acute respiratory syndrome coronavirus-2 (SARS-CoV-2), coronavirus disease 2019 (COVID-19) pandemic, is how different governments and institutions interpret and apply the same scientific evidence. This impacts on how the local guidance within each country is written and passed down to health and care workers and the general public.

Although a consensus amongst experts would be ideal, this is difficult if not impossible with a new emerging pathogen, and in some aspects, such as aerosol transmission, many experts already have pre-conceived notions which have been upheld for a long time related to other pathogens [1–3]. Such preconceptions and strongly held opinions will hamper any consensus, and bias how emerging evidence is interpreted for any new pathogen.

Issues around how a new infectious agent spreads are perhaps the most predominant, as the interventions such as social distancing, lockdowns and use of personal protective equipment (PPE) can have direct and practical impact on how people live, work and study. One of the most dramatic instances of this was (and is) the ongoing advice over the wearing of face masks.

Face masks have been used to some extent in Asia for many years to protect against inorganic airborne pollutants [4–6], so the extension to wearing masks to protect against airborne infection was relatively easy. The key aspect here is how quickly the universal masking was adopted in many Asian counties for this purpose – particularly those who had experienced and were hardest hit by the 2003 SARS-CoV-1 outbreaks. Such universal masking reduces cross-transmission and effectively creates a degree of herd immunity, which may explain why countries like Hong Kong, Taiwan, Vietnam, South Korea and Japan have experienced relatively few COVID-19 cases and deaths [7–12].

Thus, the impact of COVID-19 has been far less in those countries that experienced the SARS-CoV-12,003 or Middle East respiratory syndrome coronavirus (MERS-CoV) outbreaks, whose populations were already used to wearing face masks. This is in stark contrast to the countries that have not had this experience (including UK, Europe, North and South America), where such masking culture was relatively absent and the casualties from COVID-19 have been much higher [7, 8]. Of course, the control of COVID-19 in those Asian countries was not just due to mask-wearing but this was achieved in combination with other factors such as a relatively compliant populations, and the efficient, rapid roll-outs of mass testing, tracking and tracing, with prompt isolation of those infected, or the quarantining of those exposed.

Healthcare systems in many developed countries have gradually become more adapted to dealing with non-communicable chronic diseases (e.g. diabetes, chronic heart and lung disease, rheumatological conditions, dementia and Parkinson’s disease, etc.), depending mostly on effective vaccination programmes and a good supply of antimicrobials to deal with infectious diseases. Thus, they may be ill-prepared to deal with novel emerging pathogens. Yet, in such situations, as in any other walk of life, where expertise lies elsewhere, it seems sensible to seek and heed such advice - in this case, the Asian countries that had experienced the 2003 SARS-CoV-1 outbreaks, many of whom had also dealt with emerging threats from avian influenza A(H5N1), A(H7N9) and Middle East Respiratory Syndrome coronavirus (MERS) [13] .

Governments and populations in these Asian countries readily accepted that masking in public would protect everyone to some extent, despite relatively little evidence for the benefit of masks available at that time [14–16]. There was no prolonged debate about this and it was quickly adopted, presumably on the grounds of common sense, i.e. that any physical barrier across the face between you and someone else, without knowing which of you might be infected, was better than nothing. The protective effect of face masks has since been supported in a systematic review based on older and newer studies [17, 18].

In contrast, there were prolonged and at times acrimonious debates in Europe, North America and Australia about the benefits of wearing masks, first for healthcare workers and then in the community [19–21]. These debates went on for several months (during March–June 2020) amongst government ‘experts’ and within the media, causing much confusion to the general public. The debates initially started off with various statements to the effect that ‘masks don’t work – don’t buy them!’, which eventually changed to the more recent mandatory order that ‘masks must be worn in shops and on public transport’ [22–24].

But why the complete reversal of advice? The body of evidence for the protective effect of masks had not changed that much during this period (April–June 2020), but the way the existing evidence was presented and interpreted may have changed [17, 18], together with people’s perceptions of how the pandemic was progressing. It was becoming increasingly evident that hand-washing and social distancing alone was not controlling the spread. This was all coupled with additional, rapidly emerging evidence of airborne (i.e. fine particle) transmission, including opinions from scientists, many of whom were frequently interviewed in the media on this topic. One of the last vestiges of resistance to the benefits of masking, based on the risk compensation hypothesis (that masking makes people over-confident and take more risks), which had never been proven by any objective evidence, was also finally rebuffed [25].

There were other less dramatic examples of such reversals based on ‘mounting evidence’, such as whether or not asymptomatic SARS-CoV-2 infections truly existed - then whether or not they could transmit the virus [26–28]; and whether SARS-CoV-2 was airborne or not - and whether it could be transmitted by exhaled aerosols [29–32]. All of these possible means of SARS-CoV-2 transmission (asymptomatic and airborne) have now been acknowledged and accepted by the World Health Organization [33].

One problem is that many of these questions were initially presented in a very binary way, i.e. that some things work or don’t work, or some things happen or don’t happen, when in fact some things do work to some degree (like masks and antibody tests), and some things can occur to some extent (like asymptomatic and airborne transmission) in different circumstances. So it is perhaps not surprising that eventually some degree of reversal was inevitable. While it is understandable that government and public health guidance endeavours to be clear and concise, this is rarely possible with an emerging pathogen with limited data available. Thus, there is always the risk that an overly emphatic message may be eventually proved wrong and require correction later.

Few, if any, aspects (except survival versus death) of infectious diseases are purely black and white. It has often been forgotten that the absence of evidence is not evidence of absence (no matter how loudly and widely this is proclaimed) – especially with a novel pandemic virus. If one study does not show that something happens in a particular cohort (like asymptomatic or airborne transmission) it does not necessarily mean that it is not happening in other cohorts elsewhere.

Several obvious questions arise from this – how much and what kind of evidence needs to be presented to policy-makers to support or change their guidance? Exactly because of the nature of an emerging, novel pathogen, there is no easy answer to this, but in the face of uncertainty, some general preparedness measures can still be put in place, depending on the available resources and infrastructure, e.g. the stockpiling of PPE, the identification, enhancement and expansion of existing capacity in diagnostic laboratories, as well as isolation, quarantine and intensive care facilities.

Some of the best epidemiological modelling studies have been performed in Hong Kong since the SARS-CoV-12,003 outbreaks. One such study, published in the early stages of the Wuhan novel coronavirus outbreak, predicted the potential for widespread transmission, globally, if quick and drastic action was not taken:

*“On the present trajectory, 2019-nCoV could be about to become a global epidemic in the absence of mitigation …*. *Should containment fail and local transmission is established, mitigation measures according to plans that had been drawn up and executed during previous major outbreaks, such as those of SARS, MERS, or pandemic influenza, could serve as useful reference templates.”* (published online 31 January 2020, The Lancet) [34].

However, this produced relatively little response in the UK and other European countries [35]:

*“The NHS has been wholly unprepared for this pandemic. It’s impossible to understand why.”*

Unfortunately, by then it was already too late to prevent cases of the novel coronavirus being imported into Europe. A week earlier (24 January 2020) the first three known cases had already been detected in France [36], with 2 cases confirmed in the UK by end of January 2020 [37]. Thereafter, multiple cases of COVID-19 were reported throughout Europe [38, 39]. Yet it took another 7 weeks, despite rapidly increasing numbers of cases before there was a national lockdown on 17 March 2020 across France [40].

Hindsight is a wonderful thing, yet it is acknowledged that clear, decisive interpretations of emerging evidence for a novel pathogen will always be difficult. Early evidence will be rarely definitive from a limited number of studies, but in fact we make this kind of judgement all the time as individuals, e.g. when we buy various forms of insurance. Although certain types of insurance (like for car and buildings) are mandated by law, other forms of insurance like contents and travel insurance are really down to individual choice – which will depend on their affordability as well as that individual’s risk assessment.

So, with hindsight, could an earlier more drastic lockdown or mandated masking approach (e.g. when in crowded, essential indoor spaces, like supermarkets for food shopping) have reduced the eventual COVID-19 case numbers and deaths? Recent modelling studies in the USA along these lines suggest that this was likely, where earlier interventions could have saved 30,000–40,000 American lives [41, 42]. This could also be true of other countries.

So what factors inhibit the rapid and drastic decision-making to implement such necessary, draconian infection control measures? Many separate factors may hinder this, such as the familiarity with and the availability of masks, the significant impact of lockdown on social and economic well-being, and school and university education, etc. When experiencing such events for the first time, an interval is needed to process that information before coming up with a viable action plan that takes into account all angles unique to that population. Yet such delays only allow the virus to spread further. Even New Zealand, which has been one of the most successful countries in limiting the transmission of SARS-CoV-2, took a month after its first diagnosed case of COVID-19 to impose its strict national lockdown [43]. In a sense, each country may have to reinvent its own, unique wheel, the first time such events are encountered – thus giving the virus additional time to spread and seed greater numbers in the population.

Yet, things can improve with practice. Those countries with the experience of dealing with such epidemics of novel infectious agents will be more familiar with the need to adapt and make dramatic decisions quickly [7, 8, 44], and can offer advice to others that are willing to listen.

This advice could be related to the nature of preparedness, such as setting up rapid pathways for enhanced hospital and community-based mobile and drive-thru testing, across multiple laboratories and sites (i.e. not to keep it centralised); to develop and test mobile phone apps for enhancing track-and-trace measures; optimising existing infrastructure and facilities to enable the rapid expansion of isolation, quarantine and intensive care capacities at short notice. These preparedness capabilities will have to be developed, maintained and practiced in ‘peace’ time between pandemics, and will, inevitably, incur an ongoing cost.

Even experienced countries will need time to adapt to a new pathogen, so novel infectious agents will always be one step ahead of us, and casualties will be initially inevitable. Yet with a more coordinated international approach, an awareness of pre-conceived notions and barriers that need to be overcome, greater improvements in rapid diagnostics, epidemiological modelling, infection control, drug and vaccine development technologies, as seen since SARS-CoV-2, 2003 we will further reduce these delays before taking informed and effective action.
