# Publisher Correction to: COVID-19: interpreting scientific evidence – uncertainty, confusion and delays

**DOI:** 10.1186/s12879-020-05431-7

**Published:** 2020-09-23

**Authors:** Julian W. Tang

**Affiliations:** Department of Respiratory Sciences, University of Leicester, Leicester, LE1 7RH UK

**Correction to: BMC Infect Dis (2020) 20:653**

**https://doi.org/10.1186/s12879-020-05387-8**

Following publication of the original article [1], the author identified three errors in the article body:

- The typo “SARS-CoV-12,003”, should be written as ‘SARS-CoV-1, 2003’:
  i. in the 1st page, right-hand column, 2nd paragraph and
  ii. in the 2nd page, right-hand column, 4th paragraph;
- In the last paragraph “SARS-CoV-2, 2003” should be written as ‘SARS-CoV-1, 2003’.

The typo has been updated above and the original article [1] has been corrected.

The publisher apologizes for the inconvenience caused to the author and readers.
